# Extraction of Fucoxanthin from Raw Macroalgae excluding Drying and Cell Wall Disruption by Liquefied Dimethyl Ether

**DOI:** 10.3390/md12052383

**Published:** 2014-04-30

**Authors:** Hideki Kanda, Yuichi Kamo, Siti Machmudah, Wahyudiono, Motonobu Goto

**Affiliations:** 1 Department of Chemical Engineering, Nagoya University, Furo-cho, Chikusa-ku, Nagoya 464-8603, Japan; E-Mails: kamo.yuuichi@d.mbox.nagoya-u.ac.jp (Y.K.); machmudah.siti@d.mbox.nagoya-u.ac.jp (S.M.); wahyudiono@b.mbox.nagoya-u.ac.jp (W.); 2 Japan Science and Technology Agency, Chiyoda, Tokyo 102-0076, Japan; E-Mail: mgoto@nuce.nagoya-u.ac.jp; 3 Department of Chemical Engineering, Sepuluh Nopember Institute of Technology, Kampus ITS Sukolilo, Surabaya 60111, Indonesia

**Keywords:** dimethyl ether, supercritical CO_2_, fucoxanthin, wet extraction

## Abstract

Macroalgae are one of potential sources for carotenoids, such as fucoxanthin, which are consumed by humans and animals. This carotenoid has been applied in both the pharmaceutical and food industries. In this study, extraction of fucoxanthin from wet brown seaweed *Undaria pinnatifida* (water content was 93.2%) was carried out with a simple method using liquefied dimethyl ether (DME) as an extractant in semi-continuous flow-type system. The extraction temperature and absolute pressure were 25 °C and 0.59 MPa, respectively. The liquefied DME was passed through the extractor that filled by *U. pinnatifida* at different time intervals. The time of experiment was only 43 min. The amount of fucoxanthin could approach to 390 μg/g dry of wet *U. pinnatifida* when the amount of DME used was 286 g. Compared with ethanol Soxhlet and supercritical CO_2_ extraction, which includes drying and cell disruption, the result was quite high. Thus, DME extraction process appears to be a good method for fucoxanthin recovery from *U. pinnatifida* with improved yields.

## 1. Introduction

*Undaria pinnatifida* is consumed as one of the most popular, traditional seaweeds, particularly in East Asian countries such as Japan and Korea. In the current food industry, *U. pinnatifida* is mainly manufactured in the form of dry particles by using coarse grinder. Dry *U. pinnatifida* particles swell in hot water, and are used as an ingredient in popular foods such as bean paste soup in Japan. The main functional constituent of *U. pinnatifida* is fucoxanthin [1,2], which is a xanthophylls pigment contained in the chloroplasts of brown macroalgae. Fucoxanthin also had ability to exert effects in humans, such as antidiabetic effect [3,4], cholesterol metabolism [5,6], anti-obesity effect [1], anti-oxidant properties [2,7,8], anti-proliferative effect on cancer cells [9,10], and anti-inflammatory [11]. Extraction of bioactive compounds from macroalgae has great potential and its applications will continue to grow in the following years [12]. Pressurized solvent extraction has been used, for example, to isolate carotenoids from brown macroalgae, such as *Eisenia bicyclis* [13], *Cystoseira abies-marina*, and *Himanthalia elongata* [14]. The results showed that ethanol at high temperatures provide high recoveries of fucoxanthin and other oxygenated carotenoids. Moreover, many studies of biomaterials production have reported that cell disruption is a significant important factor to extract organic components contained in cells. For example, microwave assisted extraction of fucoxanthin from *U. pinnatifida* cells [15,16,17,18]. However, fucoxanthin exhibited sensitivity towards some factors such as light and pH, the least stable in acidic pH condition and higher concentration of ascorbic acid supplementation exerted stabilization role on fucoxanthin [19], and carotenoids are thermally decomposed in hot-drying.

Liquefied DME was used as an extractant to enhance extraction of fucoxanthin from *U. pinnatifida*. DME is the simplest form of ether [20], with the following characteristics. (i) DME has a low normal boiling point (−24.8 °C) [21], therefore, DME is not present in the final products at normal temperatures; (ii) Relative permittivity of DME is 1.08 and 5.34 at 30.5 °C, in gaseous and liquid states, respectively. Liquefied DME has high affinity to oily substances [22] and partial miscibility with water [23]; (iii) DME has been approved as a safe extraction solvent for the production of foodstuff and food ingredients by the European Food Safety Authority (EFSA) [24], by the Food Standards Australia New Zealand, and by the United States [25]. The panels of EFSA consider the intended use of dimethyl ether as an extraction solvent to remove fat from animal protein raw materials. Considering (a) that the defatted animal protein is submitted to vacuum which assures that most of the volatile dimethyl ether is eliminated from final animal protein products (b) that the maximum residual limit of dimethyl ether is of 9 μg/kg of extracted animal proteins and (c) that these proteins are used at a level of up to 2% in the final food, the Panel considered that there is no safety concern [24]; (iv) DME exhibits resistance to autoxidation, unlike other alkyl ethers [26]. Owing to these characteristics, the authors successfully extracted lipids and water from wet vegetal biomasses by liquefied DME. In the DME-based extraction technique, drying, cell disruption, and heating of solvent were not needed to improve extraction yields [27].

In this work, the effect of pressure, temperature, and ethanol as co-solvent on the recovery of fucoxanthin from *U. pinnatifida* using supercritical CO_2_ would be also presented. However, the lipids content in the extract is not presented, although supercritical CO_2_ is well known as an effective technique to extract lipids. Several experimental studies of extraction by supercritical CO_2_ have reported that supercritical CO_2_ extraction is an environmentally-friendly for lipids and carotenoids extraction from micro- and macroalgae. Mendes *et al.* carried out supercritical CO_2_ extraction of carotenoids and other lipids on whole and crushed *Chlorella vulgaris* [28]. Machmudah *et al.* and Kitada *et al.* also extracted carotenoids and astaxanthin from microalgae of *Haematococcus pluvialis* [29] and *C. vulgaris* [30] with supercritical CO_2_. The results showed that supercritical CO_2_ is more selective for carotenes than the usual organic liquid extraction and it is preferred for handling temperature-sensitive molecules. Extraction using supercritical CO_2_ with ethanol as the co-solvent has also been performed with brown seaweed at 0.8 to 30 MPa and 303 to 333 K [31]. The significant amount of oil from brown seaweed was extracted within 50 min. Roh *et al.* reported that the oil extraction yield of brown seaweed was high at higher pressure, and the amounts of fucoxanthin extracted at 20 MPa of CO_2_ pressure and 323 K of extraction temperature showed much higher than other extraction conditions [31]. As a comparison, the extraction yield from *U. pinnatifida* was also tested by using the conventional solvent ethanol with a Soxhlet extractor. As explained before that fucoxanthin has been implicated as important dietary nutrients having antioxidant potential. It is one of the reasons why some researchers had evaluated the *in vitro* antioxidant activity of fucoxanthin extracted from algae. For example, Roh *et al.* suggested that fucoxanthin content should be considered as an important feature of brown seaweed, as some of its nutritive and pharmacological effects could be attributed to their presence in plant material [31]. Billakanti *et al.* also reported that the yield of fucoxanthin from *U. pinnatifida* could be improved by using an enzyme-assisted extraction process followed by DME extraction. The results showed that extraction of fucoxanthin from *U. pinnatifida* using supercritical CO_2_ with ethanol as a co-solvent and enzymatic pre-treatment followed by DME extraction have no effect on the fucoxanthin properties [20]. Therefore, in this study the antioxidant activity of fucoxanthin was not conducted. Isolation and purification of fucoxanthin from extraction products were also not carried out.

## 2. Results and Discussion

### 2.1. Ethanol Soxhlet Extraction

It was well known that the extraction of organic compounds using a range of organic solvents from matrices (soils, sewage sludges, vegetables, and plants) has historically been carried out by using Soxhlet extraction. The apparatus for Soxhlet extraction consisted of a solvent reservoir, extractor body, an electric heat source and a water-cooled reflux condenser. Since the temperature plays a significant role for extraction, increased temperatures can disrupt the strong solute-matrix interactions caused by van der Waals forces, hydrogen bonding, and dipole attractions of the solute molecules and active sites on the matrix [32]. Therefore, the use of ethanol as an extract solvent at elevated temperature is enhanced solubility and mass transfer effects, and disruption of surface equilibria. Kim *et al.* reported that ethanol provided the best fucoxanthin extraction yield from *Phaeodactylum tricornutum* (15.71 mg/g freeze-dried sample weight) [33]. They explained that fucoxanthin content in the extracts produced by the different methods was quite constant (15.42–16.51 mg/g freeze-dried sample weight) but increased steeply based on the percentage of ethanol in water, emphasizing the importance of ethanol in the extraction. Kanazawa *et al.* also reported that 191 μg/g wet sample weight was extracted from *Laminalia japonica* with ethanol at 40 °C [34].

In this work, by using ethanol solvent, 50 μg of fucoxanthin has been extracted from U. pinnatifida 6 g. This result might be due to some contribution from the physical properties of *U. pinnatifida*. The size of *U. pinnatifida* was 0.5–3 cm directly fed without pre-treatment. The particle size is other factors that high influence the mass transfer during Soxhlet process. Bigger particles present lower ratios of surface area to volume. Conversely, smaller particles present higher ratios of surface area to volume, which enhance the contact between solvent and solid matrix and diminish the diffusion path of the particle to reach the surface, resulting in a high extraction yield. In addition, there was possibility that the degradation of fucoxanthin in ethanol solution might have occurred during Soxhlet extraction process (12 h).

### 2.2. Liquefied DME Extraction

**Figure 1:**
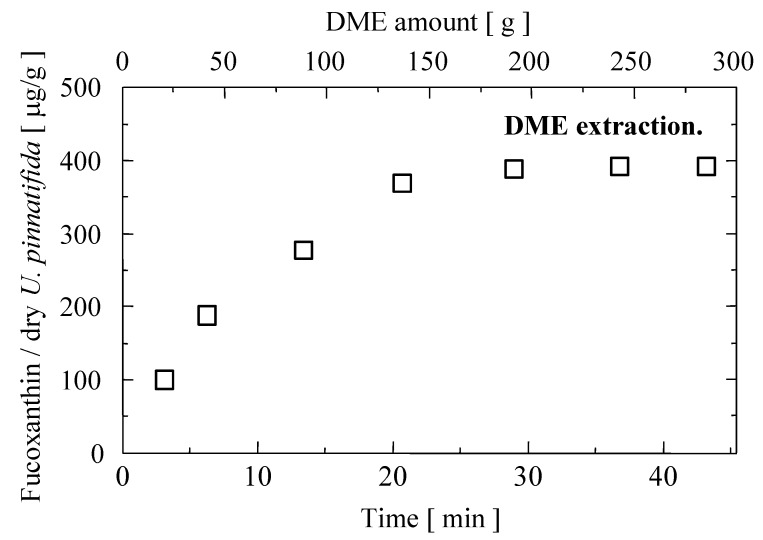
Yield of fucoxanthin in the extract obtained from wet *U. pinnatifida* by liquefied dimethyl ether (DME).

As shown in Figure 1, fucoxanthin has been extracted from *U. pinnatifida* with small amount of DME (24 g). It should be noted that the lipids content in the extract is not determined. The increasing amount of DME used was followed by increasing the amount of fucoxanthin extracted. When the amount of DME used was 286 g (at the last time point of extraction process), the residue of the *U. pinnatifida* was almost perfectly dewatered as like as such kind of dry paper. The color of *U. pinnatifida* was also changed to light green color. The amount of fucoxanthin could approach up to 390 μg/g dry of *U. pinnatifida* at this point.

Compared with other, ethanol soxhlet, the fucoxanthin amount by liquefied DME was quite high. It is well known that liquefied DME could dissolve a wide range of polar and non-polar substances. They are also good solvents for many hydrogen-bonded substances. To dissolve hydrogen bonded substances, high solvation energy was needed to break the hydrogen bonds. In this case, DME has ability to act hydrogen bond acceptors, forming hydrogen bonds with hydrogen-bonding solutes. Therefore, liquefied DME can enter into *U. pinnatifida* cells and goes out together with *U. pinnatifida* components include fucoxanthin. In addition, the cumulative fucoxanthin extracted looks like complete after 191 g of DME consumption, however, the cumulative fucoxanthin extracted seemed clearly to be enhanced with increasing the DME consumption. As mentioned before, despite liquefied DME had high dissolving ability, liquefied DME could also generate a lower viscosity of the analytes in the matrix and, accordingly, a better diffusion rate of the solute from the solid phase to the solvent. Consequently, the fucoxanthin in *U. pinnatifida* might be extracted easily.

### 2.3. Supercritical CO_2_ Extraction

**Figure 2:**
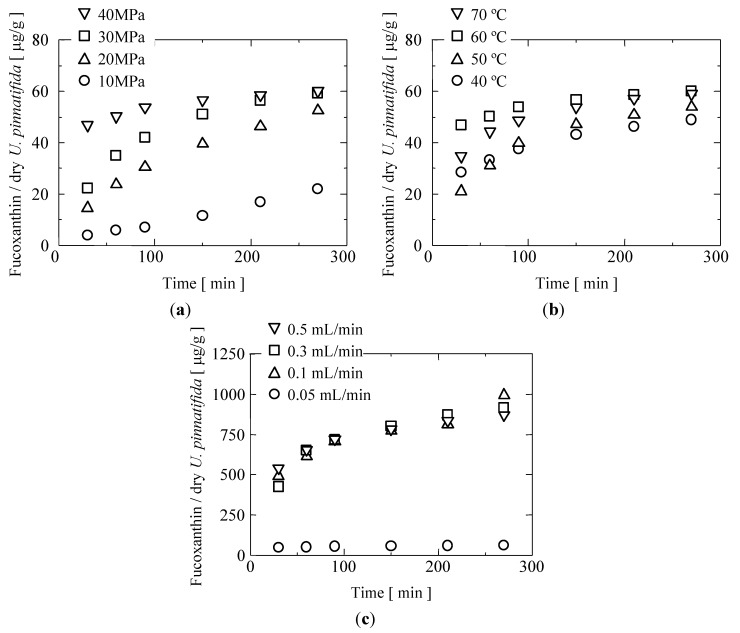
(**a**) Effect of pressures on the amount of fucoxanthin as a function of time at 60 °C of extraction temperature; (**b**) Effect of temperatures on the amount of fucoxanthin as a function of time at 40 MPa of extraction pressure; (**c**) Effect of entrainer flow rate on the amount of fucoxanthin as a function of time at 60 °C and 40 MPa.

For supercritical CO_2_ extraction system, the extraction of fucoxanthin was performed in a semi-continuous flow-type. Physically, the appearance of the extracts was oily. In general, they increased with the increase of pressure at constant temperature. This tendency happened due to direct increase of density and hence solvating power of supercritical CO_2_ [35,36]. Figure 2a,b showed the extraction curves of fucoxanthin from *U. pinnatifida* for different extraction times and under different conditions in terms of pressure (10–40 MPa; 60 °C) and temperatures (40–70 °C; 40 MPa). Initially, the yield of fucoxanthin increased significantly up to 150 min extraction time. For example, the yield of fucoxanthin is about 47 μg/g dry weight of *U. pinnatifida* dried after 30 min at 40 MPa and 60 °C. Then, it increased gradually to 58 μg/g dry weight of *U. pinnatifida* at 150 min extraction time. Over 150 min extraction time, the yield of fucoxanthin was not increase significantly. At the same extraction temperature, the same trend was found at 10, 20, and 30 MPa. As previously mentioned, the effect of pressure can be attributed to the increase in solvent power and by the strengthening of intermolecular physical interactions. It is also evident that an increase in pressure had effect on the solubility of fucoxanthin in supercritical CO_2_. This result is in agreement with similar trends reported by other authors [29,37]. Machmudah *et al.* reported that the total extract and the astaxanthin extracted were not significantly affected by the increasing pressure at 30–50 MPa, and after which the dramatic increase was observed at the pressure of 55 MPa. They explained that the dependency on the pressure was expected as the CO_2_ density increases at higher pressure, and therefore the solvent power to dissolve the substances increases [29]. At constant pressure (40 MPa), the yield of fucoxanthin also increased with increasing extraction temperature. It increased rapidly till 150 min extraction time then remained constant after 200 min extraction time in each extraction temperature. The highest extraction rate of fucoxanthin was reached when the extraction temperature is 60 °C. The maximum fucoxanthin content obtained was 60 μg/g dry weight of *U. pinnatifida* after 270 min extraction time. As shown in the Figure 2b, the amount of fucoxanthin increased with an increase in temperature. These results indicated that the fucoxanthin extractions are dependent on solute vapor pressure which increased with an increase in temperature. Instead of that, the increasing temperature contributed to the decomposition of cell walls, and as a result fucoxanthin and extractable compounds availability for extraction was increased [29]. In addition, due to the fast extraction rate of fucoxanthin from *U. pinnatifida*, this figure also showed that an increase in temperature likely had a stronger effect on the solubility than an increase in pressure. At 70 °C, the yield of fucoxanthin is lower than at 60 °C. In this case, degradation of fucoxanthin might also take place, considering that carotenoids in general are considered to be heat-sensitive [33].

In this work, the amounts of fucoxanthin extracted by supercritical CO_2_ extraction with and without ethanol as entrainer were compared. The experiments were conducted under 60 °C and 40 MPa with various entrainer concentrations (1.64%–14.29% in volume). This condition was selected because at that condition the extraction rate of fucoxanthin was high. When the concentration of 1.67% ethanol was employed, there was no significant effect on the amount of fucoxanthin extracted. As shown in Figure 2c, the enhancement of ethanol concentration had high effect on the amount of fucoxanthin extracted. The recovery of fucoxanthin could reach 10-fold. This considerable increase in extraction efficiency was because the ethanol added could enhance the solvent power of supercritical CO_2_ and caused swelling of the matrix, thus increasing the internal volume and the surface area for the contact with supercritical CO_2_ [29]. Machmudah *et al.* explained that the addition of ethanol in supercritical CO_2_ also could cause decomposition of the *H.* *pluvialis* cellular wall, with the result that the astaxanthin availability for extraction increased [29].

### 2.4. Comparison of the Results of Three Extraction Techniques

As shown in Table 1, different extraction techniques, such as soxhlet, DME, and supercritical CO_2_ extraction have been used to isolate fucoxanthin from the *U. pinnatifida* plant, however none of them can be considered as an optimal method for this purpose. Except DME extraction technique, pre-treatment of *U. pinnatifida*, such as drying, was needed. The main disadvantages of soxhlet extraction are the long time required and the large amount of solvent wasted, which is costly and cause environmental problems. Due to the long extraction time at the boiling point of the ethanol solvent, the possible degradations of fucoxanthin due to local overheating effects might occur. This process was performed to disrupt the cell membrane in *U. pinnatifida*, thus extraction efficiency of fucoxanthin increases. Supercritical CO_2_ as known is attractive extraction method for food industries because the solvent is safe, nontoxic and easily removable, the method is fast and extraction parameters can be changed in a wide range of pressure and temperature. However, CO_2_ is non-polar fluid and it is the main disadvantage in its use for the isolation of antioxidants. As explained before, to improve the fucoxanthin yield, ethanol was introduced to increase the polarity of CO_2_. However, respecting to separation process, it is not only expensive but also difficult. On the contrary, the cell disruption of *U. pinnatifida* is not required when the DME was employed as extractant due to the properties of DME (see “Introduction”). That is why this technique of fucoxanthin extraction is very simple and versatile.

**Table 1:** Best recoveries of fucoxanthin obtained using different extraction techniques.

Extraction Techniques	Time (h)	Temperature (°C)	Pressure (MPa)	Yield of Fucoxanthin (μg/g)
Ethanol soxhlet	12	78	*	50
Liquefied DME	0.72	25	*	390
Supercritical CO_2_	3	60	40	60.12
	3	70	40	59.51
Supercritical CO_2_ with entrainer (3.23%)	3	60	40	994.53

* not determined.

## 3. Experimental Section

### 3.1. Materials

**Figure 3:**
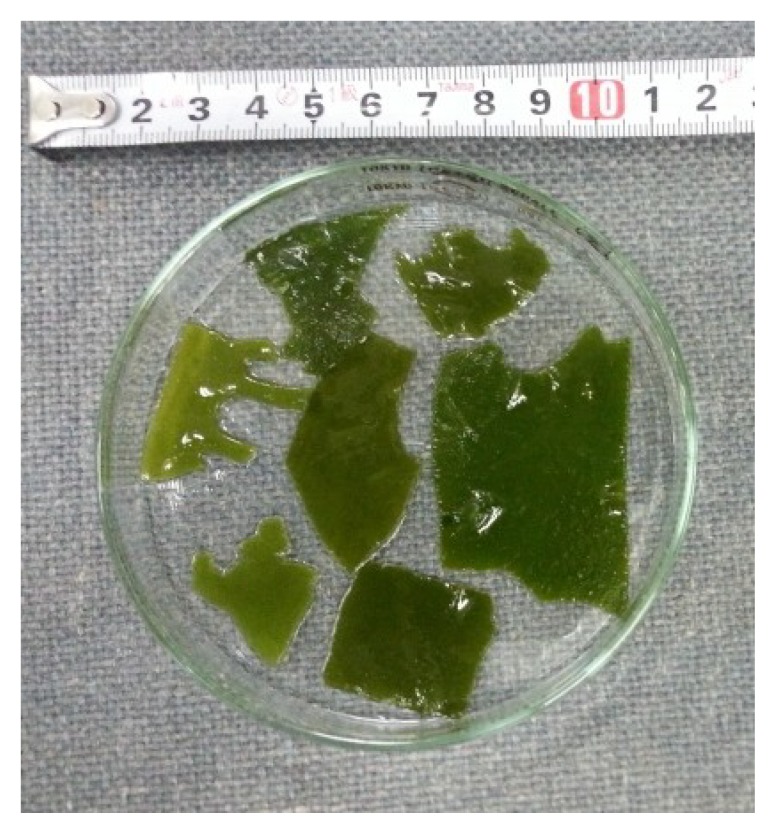
Wet *U. pinnatifida* sample.

*U. pinnatifida* was obtained from Fukui Prefecture, Japan and used as a starting material. Most of *U. pinnatifida* that did not meet strict quality standards are usually discarded as wastes, a situation, which is becoming an environmental concern. The appearance of *U. pinnatifida* is shown in Figure 3. The elemental composition of *U. pinnatifida* is given in Table 2. The apparatus of elemental analysis was conducted by a CHN analyzer (MT-6 Elemental analyzer, Yanaco New Science Inc., Kyoto, Japan) based on flash combustion, which converts all organic substances into combustion gases (H_2_O, CO_2_, N_2_). DME and CO_2_ were purchased from Tamiya, Inc. Japan and Sogo Kariya Sanso, Inc. Japan, respectively. Fucoxanthin (94.0%) purchased from Wako Pure Chemicals Industries Ltd. (Osaka, Japan) was used as standard. It was stored at −60 °C in freezer (NF-75SF3, Nihon Freezer Co. Ltd., Tokyo, Japan). The analytical reagents used were acetonitrile, chloroform, and ethanol (HPLC-grade) from Wako Pure Chemicals Industries Ltd. (Osaka, Japan). All chemicals were used as received.

**Table 2:** Elemental analysis of *U. pinnatifida* by CHN analyzer, on average.

Materials	Ultimate Analysis (wet weight%, DAF)			
	C (±0.2)	H (±0.1)	N (±0.1)	O * (±0.3)
Original algae	33.0	5.5	3.5	58.0
Residue	36.1	5.9	4.7	53.3
Extract	55.6	8.6	1.4	34.4

* Oxygen content was determined by difference.

### 3.2. Analytical Methods

**Figure 4:**
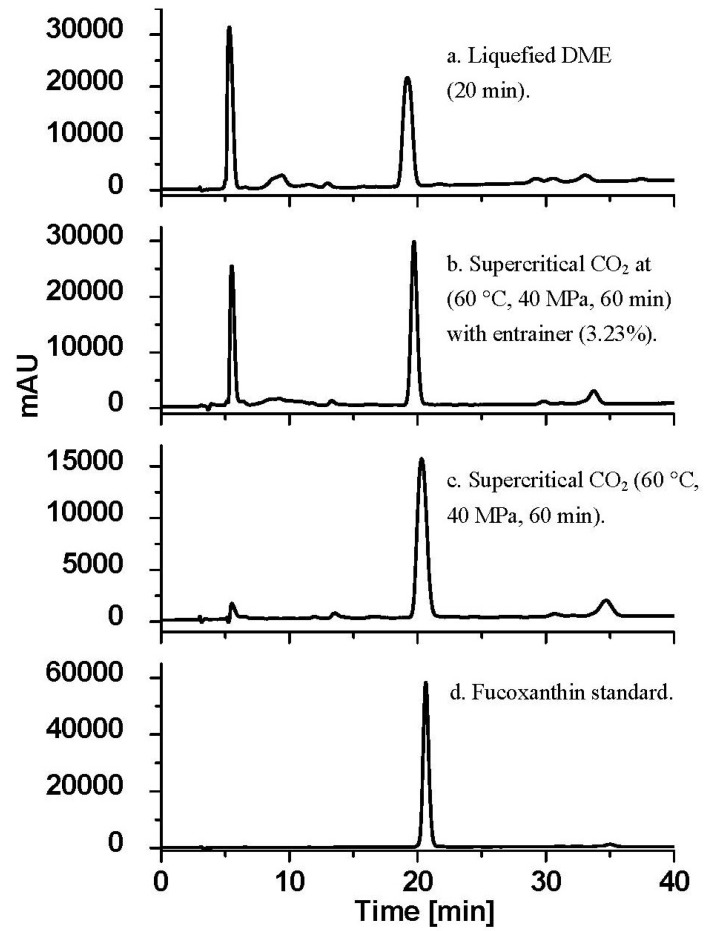
HPLC chromatogram of fucoxanthin extract from *U. pinnatifida*.

There are many carotenoids could be extracted from plant material. However, in this work, fucoxanthin was subjected as the target of carotenoid compounds recovery from *U. pinnatifida*, and determined quantitatively by using HPLC (high-performance liquid chromatography). The organic components in extracts were recovered with 2 mL of chloroform, and all the solutions were filtered using a disposable filter of 0.45 μm pore size prior to HPLC analysis. The separation of fucoxanthin was carried out according to a previous published method [30] with modification. Initially, the pure compound of fucoxanthin dissolved in chloroform as a standard was injected in the HPLC system to construct calibration curve in 5 point. After separation process in the HPLC column, the amount of fucoxanthin leaving the column will determine the intensity of the signal produced in the detector. By comparing the time it takes for the peak to show up (the retention time) with the retention times for fucoxanhin, the amount of fucoxanthin in the extract can be identified (Figure 4). This analysis can be performed with good precision; therefore, other techniques analysis was not conducted. The HPLC instrument used was an ultraviolet–visible spectroscopy detector (UV-970; JASCO, Tokyo, Japan) equipped with an ODS-3 column (GL Sciences, Tokyo, Japan; 5 μm; 250 mm × 4.6 mm) and operated at 40 °C. The mobile phase contained acetonitrile and water (80/20, v/v) with flow rate of 1.0 mL/min. Preparation of standard curve and detection were accomplished at wavelength of 445 nm.

### 3.3. Ethanol Soxhlet Extraction

In this extraction, the heating mantle temperature was set at 78 °C for 12 h. The amount of dried *U. pinnatifida* and ethanol used in flask were 6 g and 200 mL, respectively. The flask was then removed from the mantle, and the liquid extracts were transferred to bottle that covered with aluminium foil and refrigerated until analysis. In this work (DME extraction, supercritical CO_2_ extraction and ethanol soxhlet extraction), the *U. pinnatifida* was fed without any pre-treatments such as microparticulation.

### 3.4. DME Extraction

**Figure 5:**
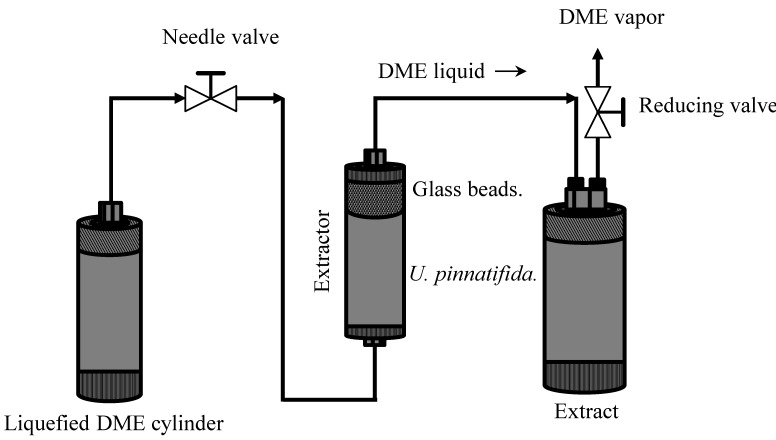
Schematic diagram of DME extraction.

Figure 5 depicted the apparatus scheme that used to evaluate the extraction efficiency of the DME extraction. The main apparatus consisted of extractor (HPG-10-5; Taiatsu Techno Corp., Saitama, Japan; volume: 10 cm^3^), needle valve to control the DME flow rate, and extract storage tank (HPG-96-3; Taiatsu Techno Corp., Saitama, Japan; volume: 96 cm^3^). Briefly, they were connected in series included feed materials. The extractor and DME storage tank were made of pressure-resistant glass coated with polycarbonate. 4.40 g of the raw *U. pinnatifida* (water content: 93.2%) was loaded into the lower half of the extractor, and the upper half was loaded with colorless glass beads. The DME flow rate was 10 ± 2 cm^3^ min^−1^. The extraction temperature and absolute pressure were 25 ± 1 °C and 0.59 ± 0.02 MPa, respectively. After passing liquefied DME through the extractor at different time intervals, the DME was evaporated by opening the reducing valve of the storage vessel. Next, liquefied DME pass through the lower half of extractor, color of glass beads in the upper half was changed to olive-green color. Because the glass beads and liquefied DME were colorless, the olive-green color indicated the probable presence of fucoxanthin. The extraction test was finished when the color of glass beads was changed again to colorless. The total time needed was less than 43 min.

### 3.5. Supercritical CO_2_ Extraction

**Figure 6:**
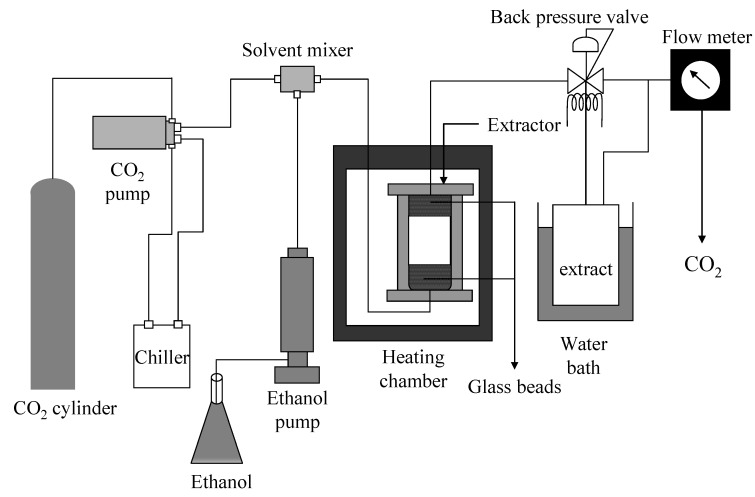
Schematic diagram of supercritical CO_2_ extraction.

The supercritical CO_2_ extraction was carried out in an apparatus with schematic diagram as shown in Figure 6. The apparatus includes a high-pressure pump for CO_2_ (PU-2086; Jasco, Hachioji, Japan), a heating chamber (WFO-400; EYELA, Tokyo, Japan), a 25 mL extraction cell (Thar Technologies, Inc., Pittsburgh, PA, USA) and back pressure regulator (AKICO, Tokyo, Japan). In this work, the extraction of fucoxanthin from *U. pinnatifida* by supercritical CO_2_ was conducted at temperatures of 40–70 °C and pressures of 20–40 MPa using a semi-continuous flow-type system with CO_2_ flow rate of 3 mL min^−1^. In each experiment, 3.0 g of dry *U. pinnatifida* sample was loaded into the extraction vessel, filled with glass beads at the bottom and top of the extraction vessel. The extraction vessel was placed in the heating chamber to maintain the operating temperature. The extracts were collected every 1 h for 5 h, weighed and analyzed immediately after collection. In all experiments, including ethanol soxhlet extraction and DME extraction, the extraction products were directly stored in the refrigerator at −60 °C. The bottles used for the collection of extracts were wrapped in aluminum foil. These processes were maintained until analysis. Co-solvent effect of ethanol on the supercritical CO_2_ extraction was also examined. Ethanol flow rates that supplied by using high-pressure pump (PU-2080; Jasco, Hachioji, Japan) were between 0.05 and 0.5 mL min^−1^. In the case of co-solvent used, the pressure and temperature selected were 40 MPa and 60 °C, respectively.

## 4. Conclusions

Liquefied DME extraction of fucoxanthin from *U. pinnatifida* was studied at temperature of 25 °C and pressure of 0.59 MPa using a semi-continuous flow-type system, a simple and environmentally friendly extraction method. Under these conditions, DME has ability to act hydrogen bond acceptors, forming hydrogen bonds with hydrogen-bonding solutes. Therefore, liquefied DME can enter into *U. pinnatifida* cells and goes out together with *U. pinnatifida* components include fucoxanthin. The amount of fucoxanthin could approach to 390 μg/g dry *U. pinnatifida* when the amount of DME used was 286 g. Compared with supercritical CO_2_ and ethanol Soxhlet extraction, the result was quite high. Thus, DME extraction process appears to be a good method for fucoxanthin recovery from *U. pinnatifida* with improved yields. On the basis of these results, it is proposed that DME extraction is applicable to isolate carotenoids from other types of biomass.
